# Clinical and Lab profile of severe and uncomplicated malaria: A prospective study from Khuzdar Balochistan

**DOI:** 10.12669/pjms.37.7.4210

**Published:** 2021

**Authors:** Memoona Khan, Haider Nisar, Nuzhat Mushahid

**Affiliations:** 1 Dr. Memoona Khan, MBBS, FCPS Haematology, Consultant Haematologist, Combined Military Hospital Khuzdar, Khuzdar District Balochistan, Pakistan; 2 Dr. Haider Nisar, MBBS, FCPS Paediatrics Consultant Paediatrician, Combined Military Hospital Khuzdar, Khuzdar District Balochistan, Pakistan; 3 Dr. Nuzhat Mushahid, MBBS, FCPS Haematology, Professor of Haematology, Chairperson PSH, Ex Commandant Armed Forces Institute of Transfusion, CEO, P-First Solution, Sector F, DHA-1, Islamabad, Pakistan

**Keywords:** Clinical profile, Cerebral Malaria, Plasmodium vivax, Plasmodium falciparum

## Abstract

**Objectives:**

Khuzdar, the largest city of Southern Balochistan, is endemic for malaria with API of 6. The study was aimed at comparing the clinico-lab profile of severe and uncomplicated malaria in the region and to determine any association with age. No such study is reported in the region so far.

**Methods:**

A prospective clinical observational study was conducted in Combined Military Hospital, Khuzdar between 2018 and 2020. A total of 210 Malaria patients, irrespective of age and gender were included. Cases were categorized into severe and uncomplicated according to WHO criteria. The clinical parameters and lab profile of severe and uncomplicated cases were compared and data was analyzed using SPSS 23.0. Categorical variables were analyzed for association of clinical features with severe malaria using Fisher exact test. Continuous variables were compared between uncomplicated and severe malaria using Mann-Whitney U –test. Statistical significance of lab parameters with type of malaria was derived using Kruskal Wallis.

**Results:**

Uncomplicated and severe cases were 191 (91%) and 19 (9%) respectively. Severe malaria was significantly associated with jaundice, bleeding from gums, epistaxis, pallor, vomiting, respiratory distress, thrombocytopenia, low Hb, raised serum bilirubin and raised PT (p value<0.05). In children, frequency of multiple complications was significantly higher than adult patients. (75% vs 25%, p-value 0.002).Overall case fatality of severe malaria was 5.2% (1/19). However, case fatality rate was 100% in cerebral malaria.

**Conclusions:**

Certain clinical and lab parameters can be used to predict prognosis and thus avoid adverse outcome in malaria patients.

## INTRODUCTION

Malaria remains one of the most prevalent parasitic diseases across the world, claiming about half a million deaths globally with majority occurring in Africa followed by Southeast Asia and South America.^1^ An estimated 228 million cases of malaria occurred worldwide in 2018. Although 50% of malaria cases in Southeast Asia were due to *P.falciparum*, the region contributed to 53% of *P.vivax* burden in 2018. According to WHO, Malaria Incidence rate in Pakistan in 2018 was 1-10(cases per 1000 population at risk).^2^

Pakistan is among seven countries of WHO Eastern Mediterranean Region sharing 98% of total regional malaria burden. In 2018, a total of 374,513 confirmed malaria cases were reported from all health facilities to Federal Directorate with 16.4% (61,510) cases from Balochistan. *P.vivax* accounted for 84% of all malaria cases. Lack of access to healthcare facilities/low blood examination rates may be the reason of low reported cases in Balochistan.^3^ Balochistan province among other provinces of Pakistan is susceptible to epidemic outbreaks and transmission of malaria species varies from season to season.^4^

Clinical presentation of malaria varies from asymptomatic infection to benign/uncomplicated disease and even severe/fatal cases depending on host and environmental factors.^5^ While *P.vivax* was considered to cause uncomplicated malaria previously, systematic review of clinical studies of severe malaria has shown increase in severe and even fatal cases of vivax over past 15 years.^6^,^7^ Research has shown that clinical/lab profile of disease varies from region to region and malaria complications vary substantially across the world. Certain Lab parameters may be useful to monitor evolution of disease and assess response to treatment.^8^-^11^

Most of the studies regarding clinico lab profile of malaria are restricted to areas of high endemicity like Africa^12^, whereas studies carried out in moderate endemic areas like Pakistan, are scarce. As per available data of Pakistan, coastal city of Karachi, Thatta, Badin, Zhob, Larkana and Nawabshah are considered as holoendemic for malaria^13^, available data of Balochistan reveals North Balochistan as mesoendemic and Ziarat and Kalat as hypoendemic.^14^ A few preliminary studies have been conducted in Khuzdar which mainly document the occurrence and frequency of various types of malaria. However, data comparing clinical and lab spectrum of severe and uncomplicated malaria cases in Khuzdar is not available to date. Given this scenario, this prospective study aimed at collecting information regarding spectrum of clinical and lab profile of malaria cases (vivax, falciparum and mixed) presenting to Combined Military Hospital Khuzdar.

## METHODS

The study was conducted in the department of pathology at Combined Military Hospital Khuzdar.This was a prospective clinical observational study. Non probability consecutive sampling technique was used. The study was conducted over a period of two years from October 2018 to September 2020. Approval was obtained from the hospital ethics committee for conducting the study (File No.02/ERC/CMH Khz) Dated 29 October 2020.

### Study population

Subjects of the study included all patients irrespective of age and gender, admitted with the diagnosis of *P.vivax*, *P.falciparum* and Mixed (*P.vivax* and *P.falciparum*) malaria infection. Patients with age less than 12 years were classified as paediatric population. Cases of relapsed malaria, those labeled as Clinical Malaria or Cases with any other coinfection were excluded from the study.

### Clinical Assessment

Data were collected in a tabulated form in a proforma after taking informed consent and consisted of demographic profile, detailed history, and general and systemic examination of patients. Patients were classified as uncomplicated or severe malaria cases according to WHO clinical and laboratory criteria. Severe falciparum malaria was defined as one or more of the clinical or laboratory parameters, occurring in absence of an identified alternative cause, and in the presence of P.falciparum asexual parasitemia. The criteria for severe vivax malaria were the same as for severe falciparum with no parasitaemia density thresholds^15^. Uncomplicated malaria was defined as a malaria case with symptoms such as fever>38 C, headache, chills and/or malaise with a positive peripheral smear examination +/- RDT without severity criteria irrespective of parasite species. Severe cases were further defined as having single complication or multiple (>one complication)

### Laboratory tests

Diagnosis of malaria was made on microscopy through peripheral smear examination of conventional thick and thin blood films, stained with Giemsa stain, and rapid diagnostic tests (RDTs). The Tuber^R^ Line Malaria Pf/Pv Rapid test Cassette (Whole Blood) was used. Blood samples of patients with clinical suspicion of malaria were evaluated for malarial parasite through combined peripheral film examination and RDT. The RDT is a lateral flow chromatographic immunoassay for the simultaneous detection and differentiation of *P.falciparum* and *P.vivax* antigens in whole blood. The membrane is pre-coated with anti HRP –II antibodies. During testing, the whole blood reacts with pre coated dye conjugate on test cassette, the mixture then migrates upward by capillary action, reacts with anti HRP-II on *P.falciparum* test line region or with anti *P.LDH* on *P.vivax* line region depending on whether the specimen contains HRP-II or Plasmodium-specific *P.vivax* LDH or both yielding colored lines in the specific regions.

Complete blood cell counts were performed on automated haematology analyzer (XP-100 Sysmex, Japan). Sera for biochemical parameters were run on semi-automated chemistry analyzer (Merck Microlab 300 LX). Prothrombin Time (PT) was performed manually using Helena Bioscience Europe Thromboplastin L KIT. Patients with other coinfections like typhoid and viral hepatitis were excluded from the study after appropriate testing.

### Statistical Analysis

It was performed using Statistical Package for the Social Sciences (SPSS) for Windows, Version 23.0. Chicago, SPSS Inc. Categorical variables were analyzed for association of clinical features with severe malaria using Fisher exact test. Continuous variables were compared between uncomplicated and severe malaria using Mann-Whitney U –test. Statistical significance of lab parameters with type of malaria was derived using Kruskal Wallis test. *P* ≤ 0.05, with confidence interval of 95%, was considered as statistically significant.

## RESULTS

A total of 210 admitted patients positive for vivax, falciparum and mixed (vivax and falciparum) malaria on peripheral smear examination and/or antigen-based RDT were included in this study. Descriptive statistics and frequencies of various malaria species in study population and clinical features, are shown in Table-I.

**Table I:** Characteristics of Patients (n=210).

*N*	*210*
Age (Years)	29.40±7.29
Age [Range(Years)]	3-70
Gender (Male/Female)	[190(90.5%)/20(9.5%)]
***Type of Malaria***	
Falciparum	62(29.5%)
Vivax	134(63.8%)
Mixed	14(6.7%)
***Severity of Malaria***	
Severe	19(9%)
Uncomplicated	191(91%)
***Clinical Features***	
Fever	210(100%)
Prostration	1(0.5%)
Bleeding from Gums	2(1%)
Cerebral Malaria	1(0.5%)
Hypoglycemia	1(0.5%)
Epistaxis	4(1.9%)
Pallor	25(11.9%)
Vomiting	6(2.9%)
Jaundice	10(4.8%)
Respiratory Distress	4(1.9%)

Patients with age<12 years were classified as children. Out of 210 cases, eight (3.8%) were children (mean age 8.5, range 3-11). All children presented with complications and were classified as having severe malaria.

### Clinical findings

Frequencies of clinical features were significantly higher among severe cases of malaria. (Table-II).

**Table II:** Comparison of Clinical Features in Severe and Uncomplicated cases.

	*Clinical features*	*Severe (n=19)*	*Uncomplicated (n=191)*	*p-value*
*Clinical Features*	Prostration	1(5.3%)	0(0%)	0.090^(a)^
	Bleeding from Gums	2(10.5%)	0(0%)	0.008^(a)**^
	Cerebral Malaria	1(5.3%)	0(0%)	0.090^(a)^
	Hypoglycemia	1(5.3%)	0(0%)	0.090^(a)^
	Epistaxis	4(21.1%)	0(0%)	<0.001^(a) **^
	Pallor	9(47.4%)	16(8.4%)	<0.001^(a) **^
	Vomiting	4(21.1%)	2(1%)	0.001^(a) **^
	Jaundice	10(52.6%)	0(0%)	<0.001^(a) **^
	Respiratory distress	4(21.1%)	0(0%)	<0.001^(a) **^

***Note***(a): Fisher exact test was applied to calculate p-value.

### Profile of lab findings

Descriptive statistics of lab parameters assessed for patients and correlation with parasite species are shown in Table-III. There were no significant differences between lab parameters of different species of malaria. (Table-III).

**Table III:** Comparison of Laboratory Parameters with Type of Malaria.

		*Type of Malaria*			*p-value*

		*Vivax*	*Mixed*	*Falciparum*	

		*134*	*62*	*14*	

		*Mean±SD*	*Mean±SD*	*Mean±SD*	
*Lab Profile*	Hemoglobin	13.86±1.63	13.66±1.84	13.43±2.96	***0.965***
	Platelet Count	102.43±35.49	124.28±38.34	100.48±40.11	***0.134***
	TLC	4.97±1.42	4.62±1.04	4.77±2.44	***0.168***
	T-Bilirubin	13.82±16.22	11.85±2.44	16.04±18.53	***0.245***
	PT	14.97±1.41	14.71±0.72	15.5±1.97	***0.106***
	Creatinine	91.37±10.46	94.86±10.91	93.08±15.08	***0.347***

***Note***(a):Kruskal Wallis test was applied to calculate p-value.

All lab parameters, except TLC and creatinine showed significant difference with the clinical severity. Based on these findings, the study suggests that Hb, platelet count, T-Bilirubin and PT can be used as prognostic markers for malaria. (Table-IV).

**Table IV:** Comparison of Laboratory Parameters in Severe and Uncomplicated cases.

*Respiratory Distress*		*4(21.1%)*	*0(0%)*	0.000^(a) **^

		*Severe (n=19)*	*Uncomplicated (n=191)*	*p-value*

		*Mean±SD*	*Mean±SD*	
Lab Profile	Hemoglobin ( 12.0-16.5 g/dL)	10.88±3.60	14.00±1.68	0.001^(b) **^
	Platelet Count (150-400 x 10^9^/L)	68.10±31.78	106.81±36.09	<0.001^(b) **^
	TLC (4-11 x 10^9^/l)	5.60±3.85	4.81±1.40	0.636^(b)^
	T-Bilirubin ( ≤ 17 mmol/l)	46.36±42.32	11.16±3.74	<0.001^(b) **^
	PT (control 14 s).	18.05±3.08	14.81±0.95	<0.001^(b) **^
	Creatinine (62-120 umole/l)	95.32±23.96	91.79±10.17	0.450^(b)^

***Note*** (b): Mann Whitney U test was applied to calculate p-value.

### Severe Malaria

A total of nineteen (9%) patients (78.9% male***)*** were classified as severe malaria. Fifteen patients (78.9%) had a single complication including one patient of cerebral malaria, while only four (21.05%) patients had multiple complications.

**Fig.1:**
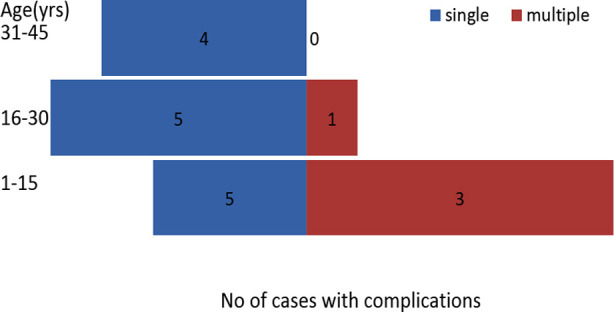
Association of complications with age group is shown.

The frequencies of clinical features in severe malaria are shown in Table-III. Only one patient of cerebral malaria died during the study period. The case fatality rate of cerebral malaria was 100% while case fatality rate in severe cases was 5.2% (1/19). Number of complications was significantly associated with age group of patients (Fig.1).

It was observed that in paediatric age group frequency of multiple complications were significantly higher as compared to adult patients (75% vs 25%) Chi square Test=8.872, p-value 0.002.

## DISCUSSION

To our knowledge, this is the first study carried out in Khuzdar on the clinical and laboratory profile of severe and uncomplicated malaria. We found that certain clinical and laboratory parameters are significantly associated with severity of the disease and can be used as prognostic markers. As compared to past, there is now sufficient evidence available in literature supporting the evidence of *P.vivax* contributing to severe malaria burden.^5^-^7^,^16^ Similar were the findings reported in our study.

We report *P.vivax* as the leading cause of malaria (63.8%) followed by *P.falciparum* (29.5%) and mixed (6.7%). This was in accordance with Pakistan Annual Malaria report 2019^3^ and also correlates with global epidemiology of *P.vivax*.^6^,^17^ Yasinzai, however, reported *P.falciparum* as the more prevalent species in Barkhan and Kohlu, bordering areas of East Balochistan.^18^ 90.5% cases were males, and the male to female ratio was 9.5:1. The male predominance was also reported in Karachi^19^, FATA areas of Khyber Pukhtunkhwah^20^ and Delhi, India.^6^

All patients presented with fever. However, symptoms of vomiting and signs of pallor and icterus were more frequently seen in patients of severe malaria. These were the only warning signs of malaria observed in our study population. Mathews SM from Delhi reported abdominal pain, headache, altered consciousness and cough with breathlessness as potential risk factors for development of severe malaria.^6^

The high prevalence of uncomplicated cases as seen in our study, which may be due to better knowledge, attitude and practices in the study population, early access to healthcare facilities, prompt diagnosis and effective management of malaria cases, was in agreement with similar studies carried out in Khuzdar between 2003 to 2004^21^ and Multan^22^ and also with a study reported by Arévalo-Herrera M in Colombia.^5^ However, Mathews SM from Delhi reported a high percentage of severe malaria cases defined by WHO criteria. While in our study, *P.vivax* contributed to 47.3% of severe malaria cases, 42% severe vivax cases were reported from a tertiary hospital in Delhi.^6^

Fever, jaundice and pallor were the most frequent complications in our study which was similar to a study done at Peshawar, where fever (100%) and pallor (50%) were also the most common presenting symptoms of children with severe malaria.^19^ A tertiary care study from Delhi also reported jaundice as the most frequent complication and other reported were ARDS (20.6%), significant bleeding (14.2%), metabolic acidosis (12.6%) and acute kidney injury and cerebral malaria 7.9% each in a total of 150 patients. As opposed to this, no case of metabolic acidosis or acute kidney injury was observed in our study. We report only one case (5.3%) of cerebral malaria as compared to five (7.9%) cases of neurological manifestations in Delhi.^6^

Hemoglobin levels, platelet count, total bilirubin and prothrombin time were significantly different among uncomplicated and severe malaria cases. However, we could not find any significant association between type of malaria species and lab profile. Contrary to this, Zubairi et al. reported bilirubin, Hb and platelet count to be significantly associated with falciparum malaria.^23^ Although pallor was noted in 47.4% of severe cases, there was no case of severe anaemia as per WHO criteria^2^ in our study. In the study conducted at Delhi, there were four (2.6%) cases of severe anaemia.^6^ In our study, thrombocytopenia (< 100 x 10^9^/l) was seen in 50.9% of all cases of malaria and severe thrombocytopenia (<50 x 10^9^/l) was seen in seven cases only. Six out of these seven cases developed severe malaria while one case had uncomplicated malaria. These findings correlated with the results of study carried out in Columbia^5^ while the study in Delhi^6^ reported thrombocytopenia in 86.7% of all cases by using a threshold of plt count< 150 x 10^9^/l. Only two patients inducted in our study, out of a total of seven with severe thrombocytopenia, presented with spontaneous bleeding. Both anaemia and thrombocytopenia could be useful lab parameters to predict severity of malaria. There are multiple factors responsible for etiology of both these complications, including immune mechanisms^24^, this hypothesis is not tested in this study.

Children comprised of 75% of study population presenting with multiple complications. Similar findings were reported in a study carried out in Karachi, which reported multiple organ dysfunction in 70% of children with severe malaria.^25^ Our finding is also in accordance with WHO report 2018, which emphasized upon the fact that in high transmission areas, most of severe malaria cases occurred in young children without acquired immunity. Both cases of hypoglycemia and cerebral malaria were also reported in paediatric age groups which is again in agreement with WHO findings.^2^ However, this conclusion could not be drawn in study carried out in Delhi as all patients included in the study were adults.^6^

The case fatality rate was 5.2% (1/19) in severe malaria cases in our study. The same, labeled as incidence of mortality, was reported to be 3.17% (2/63) in the study carried out in Delhi.^6^

### Limitations

The study was conducted in Khuzdar and included admitted malaria patients only. Therefore, the results of study are not representative of the entire Balochistan population.

## CONCLUSIONS

The study showed a high prevalence of uncomplicated malaria cases which may be associated with early diagnosis and effective management. However, paediatric population particularly remains at risk for developing severe malaria and multi organ involvement.

Moreover, the study also signifies the importance of certain clinical and lab parameters which could be used as prognostic markers for disease progression and such cases could be vigilantly monitored to avoid adverse outcomes.

### Authors’ Contribution:

**MK:** conceived, designed and editing of manuscript. She is also responsible for the accuracy or integrity of the work.

**HN:** did Data collection and manuscript writing.

**NM:** did review and final approval of manuscript.
